# PSO-LocBact: A Consensus Method for Optimizing Multiple Classifier Results for Predicting the Subcellular Localization of Bacterial Proteins

**DOI:** 10.1155/2019/5617153

**Published:** 2019-11-19

**Authors:** Supatcha Lertampaiporn, Sirapop Nuannimnoi, Tayvich Vorapreeda, Nipa Chokesajjawatee, Wonnop Visessanguan, Chinae Thammarongtham

**Affiliations:** ^1^Biochemical Engineering and Systems Biology Research Group, National Center for Genetic Engineering and Biotechnology (BIOTEC), King Mongkut's University of Technology Thonburi, Bangkhuntien, Bangkok 10150, Thailand; ^2^Food Biotechnology Laboratory, National Center for Genetic Engineering and Biotechnology (BIOTEC), 113 Phahonyothin Rd., Khlong Luang, Pathumthani 12120, Thailand

## Abstract

Several computational approaches for predicting subcellular localization have been developed and proposed. These approaches provide diverse performance because of their different combinations of protein features, training datasets, training strategies, and computational machine learning algorithms. In some cases, these tools may yield inconsistent and conflicting prediction results. It is important to consider such conflicting or contradictory predictions from multiple prediction programs during protein annotation, especially in the case of a multiclass classification problem such as subcellular localization. Hence, to address this issue, this work proposes the use of the particle swarm optimization (PSO) algorithm to combine the prediction outputs from multiple different subcellular localization predictors with the aim of integrating diverse prediction models to enhance the final predictions. Herein, we present PSO-LocBact, a consensus classifier based on PSO that can be used to combine the strengths of several preexisting protein localization predictors specially designed for bacteria. Our experimental results indicate that the proposed method can resolve inconsistency problems in subcellular localization prediction for both Gram-negative and Gram-positive bacterial proteins. The average accuracy achieved on each test dataset is over 98%, higher than that achieved with any individual predictor.

## 1. Introduction

The prediction of the subcellular localization of proteins is a significant step in protein function annotation, providing useful insights into biological functions and interactions. Information involving the subcellular localization of proteins in bacteria can support the development of drugs and vaccines [1]. Bacterial cell surfaces and secreted proteins are of interest for their potential as vaccine candidates or diagnostic targets. Using experimental techniques, identifying the subcellular localization of a protein is relatively laborious and time consuming. However, reliable and accurate computational methods of predicting subcellular localization can accelerate this process. Over the past decades, numerous prediction methods have been proposed as a result of independent efforts by various research teams (summarized in Table 1). Yu et al. [2, 3] developed CELLO, a multilayered SVM classification system that uses 4 types of sequence coding schemes, namely, amino acid composition, dipeptide composition, partitioned amino acid composition, and physicochemical-property-based sequence composition, to predict protein locations. Bhasin et al. [5] developed PSLpred, which includes various SVM modules based on features such as amino acid composition, dipeptide composition, physicochemical properties, and evolutionary information from PSI-BLAST. Later, SLP-Local [6] was developed to predict the subcellular localization of proteins based only on the local compositions of amino acids and twin amino acids and the local frequencies of the distances between successive amino acids. SOSUI-GramN [1] was proposed as a predictive software system developed specifically for assessing the subcellular localization of proteins in Gram-negative bacteria. It utilizes only the physicochemical parameters of the N- and C-terminal signal sequences and the total sequence. In particular, SOSUI-GramN offers markedly improved accuracy for the localization prediction of extracellular proteins, which is commonly known as a weakness of other methods. Gneg-mPLoc and Gpos-mPLoc were developed by Shen et al. [7, 9] as components of Cell-PLoc [8, 13], a web server for predicting the subcellular localization of proteins in various organisms. These tools can be used for cases in which a query protein may simultaneously exist in more than one location. PSORTb 3.0, the latest version of a well-known method for bacterial protein analysis [10], uses information on amino acid composition, similarity to proteins of known localization, the presence of a signal peptide, transmembrane alpha-helices, and motifs corresponding to specific locations found for each given protein to determine its subcellular localization. By using a probabilistic method, PSORTb 3.0 outperforms CELLO, Cell-PLoc, SLP-Local, and the previous versions of the same tool. King and Guda [11] proposed an n-gram-based Bayesian subcellular localization classifier called ngLOC. As part of its output, ngLOC provides a set of probabilistic scores for the top three possible locations of each given protein. Later, in early 2014, Goldberg et al. [12] presented LocTree3, a profile kernel SVM with the addition of homology-based inference, for protein subcellular localization prediction. Yu et al. [4] presented a new version of CELLO called CELLO2GO, which combines the original technique with information regarding gene ontology (GO) categories to describe the functions of genes and gene products across species.

Nevertheless, each prediction program has unique weaknesses and strengths depending on the adopted training strategies and algorithms. Specifically, these tools differ in three notable aspects: the underlying biological model, location coverage, and prediction accuracy [14]. A given tool may not be able to accurately predict the exact localization of every protein. It often happens that one predictor performs better for some cases while another predictor performs better for another compartment or under other circumstances. During the genome annotation process, a user may consider results from multiple prediction programs to confirm the final prediction and may encounter conflicting predictions. It is difficult for users to arrive at sensible decisions when faced with two or more contradictory predictions made by multiple programs [15]. To address this problem, the combination of multiple predictive models via a consensus classifier has become a promising solution. Efforts have been made to combine results from multiple predictors to generate a final prediction. In 2012, a metapredictor for protein localization in Gram-negative bacteria was introduced by Magnus et al. [16]. Their predictor combines the results from various prediction tools by using 5 one-versus-rest binary logistic regression models. This approach was developed based on the conversion of the multiclass classification problem into a set of independent binary logistic regression classification problems. On this basis, the class label corresponding to the logistic regression classifier with the highest probability will be returned as the final prediction. However, naïvely comparing the probabilities of separate and independent binary logistic regression classifiers may result in irrelevant decision boundaries that will affect the correctness of the final prediction due to imbalances between the classes. Therefore, the motivation of this work is to instead estimate the probabilities of all classes simultaneously; hence, the interdependence of all classes will also be estimated as part of the joint classification process.

To this end, we propose a new subcellular localization predictor for bacterial proteins using particle swarm optimization (PSO) that efficiently combines prediction results from preexisting predictors to improve the overall predictive accuracy and resolve incongruent results from different predictors. To date, many subcellular localization predictors have been proposed. The goal of this work is not to develop another trained classifier based on certain selected features; instead, the aim is to introduce a PSO-based consensus classifier to combine and enhance the strengths of the previous methods. The main reasons for choosing PSO instead of another optimization method for this multiclass problem are its iterative search capability for identifying the global optimum in a multidimensional space and its ease of continuous data representation, which permits easy modification in the case of removing or adding predictors. Moreover, PSO does not rely on the gradient of the problem to be optimized; thus, PSO does not require that the optimization problem be differentiable, as is required by classic optimization methods [17–19]. Recently, a PSO-based consensus method has been successfully applied to classify eukaryotic protein localization results [20].

In this work, the application of PSO in optimizing the weights and biases of various prediction methods enhances the accuracy of a prediction model for protein localization in bacterial genome sequences. This method can be used to identify the locations of the proteins from 5 locations in Gram-negative bacteria (extracellular region or secreted proteins, outer membrane, periplasm, inner membrane or cytoplasmic membrane, and cytoplasm) or 4 locations in Gram-positive bacteria (extracellular region, cell wall, inner membrane, and cytoplasm). Empirical experiments performed under various circumstances suggest that the proposed PSO-based consensus classifier offers significantly improved performance compared with the individual predictors.

## 2. Materials and Methods

**Figure 1:**
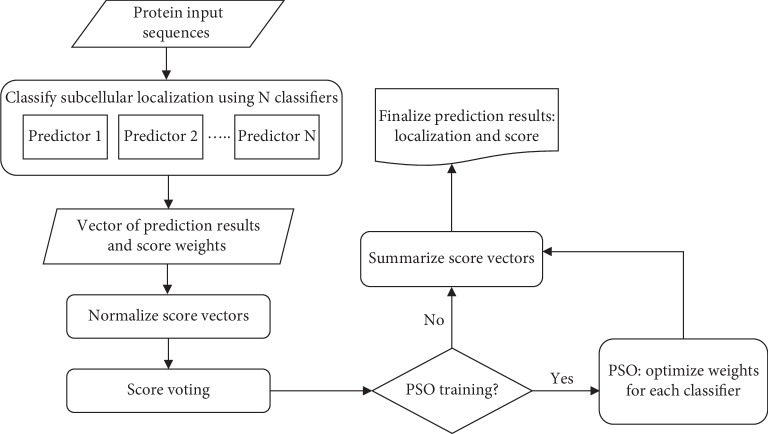
Flowchart of the proposed algorithm.

The flowchart of the proposed method is illustrated in Figure 1.

### 2.1. Data Collection

Protein sequences with known locations were extracted from UniProtKB [21]. Only sequences with the reviewed (Swiss-Prot manually annotated) status were collected. Duplicated proteins with over 90% sequence identity were removed by using CD-HIT [22]. We randomly selected 2,150 Gram-negative and 1,866 Gram-positive nonredundant bacterial proteins with less than 90% sequence identity from the resulting dataset. For each dataset, approximately 80% of the data were used as a training set, and the remaining proteins after removal were used as a test set. The test dataset for Gram-negative bacterial proteins covered five locations, with 86 proteins for each location. The test dataset for Gram-positive bacterial proteins consisted of 311 proteins, including 79 sequences from cytoplasm, 79 sequences from inner membranes, 77 sequences from cell walls, and 76 sequences from extracellular regions. After data collection, the following individual predictors for bacterial protein subcellular localization were employed as the selected classifiers: CELLO [2, 3], PSORTb 3.0 [12], CELLO2GO [4], SOSUI-GramN [1], SLP-Local [6], ngLOC [11], Gneg-mPLoc [9], Gpos-mPLoc [7], PSLpred [5], and LocTree3 [12]. Some of them are available for local standalone installation, whereas some are available only on web servers. For servers that do not accept one file containing multiple protein sequences, we used the screen-scraping technique with Python to submit inputs and fetch outputs (the screen-scraping codes are also provided with the software). CELLO, PSORTb 3.0, ngLOC, and SLP-Local yield scores for the probabilities of class assignment, whereas the other programs provide only the location predictions; hence, in the latter case, we assigned a label of 1 to the predicted location and a label of 0 to the other locations. Once all results had been obtained in the form of numerical vectors, we simply combined them into one CSV file to serve as the input for the PSO classifier.

In addition to the data described above, we also employed the benchmark dataset S taken from [7, 9]. This dataset includes 523 proteins (4 locations) for Gram-positive bacteria and 1,404 proteins (5 locations) for Gram-negative bacteria. None of the proteins included in dataset S has a pairwise sequence identity of >25% with respect to any other in the same subcellular location. This dataset S is much more rigorous in excluding homology bias and redundancy. Moreover, this dataset is well documented and has been used in benchmarking various predictors [7, 9, 23–30].

### 2.2. Experimentation

This section briefly explains the experimentation involving the performance comparison of the proposed method in different settings. All predictors were evaluated on the same test datasets. The steps of the algorithm are described below.

1. The result score matrix was prepared and loaded for the PSO classifier. This score matrix was used for weight optimization in the PSO algorithm.
2. The weights were multiplied by the scores. Scores for the same location were summed together, and the results were then sorted in descending order. The location with the maximum score was selected for comparison with the given class label of each protein sequence.
3. The performance of the method was further evaluated by considering the following 9 experimental cases:
4. The classifier with the highest accuracy was removed to observe how its removal would influence the result.
5. Tools that exhibited an accuracy lower than 90% were removed.
6. As a complement to 3.2, all other tools with an accuracy of 90% or higher were removed to determine whether the proposed method could improve the prediction accuracy in the case of only relatively inaccurate predictors.
7. Tools that exhibited an accuracy lower than 80% were removed.
8. As a complement to 3.4, all other tools with an accuracy of 80% or higher were removed.
9. Tools that exhibited an accuracy lower than 70% were removed.
10. As a complement to 3.6, all other tools with an accuracy of 70% or higher were removed.
11. Tools that exhibited an accuracy lower than 60% were removed.
12. As a complement to 3.8, all other tools with an accuracy of 60% or higher were removed.

All experimental results are reported and compared to illustrate the effects of the different settings on the proposed method. In every step of the evaluation, the overall prediction accuracy was calculated as shown in

(1) $$\begin{array}{c} \text{accuracy }\left(\text{ACC}\right)=\frac{\left|\text{number of correct answers}\right|}{\left|\text{number of instances}\right|}. \end{array}$$

### 2.3. Particle Swarm Optimization

**Figure 2:**
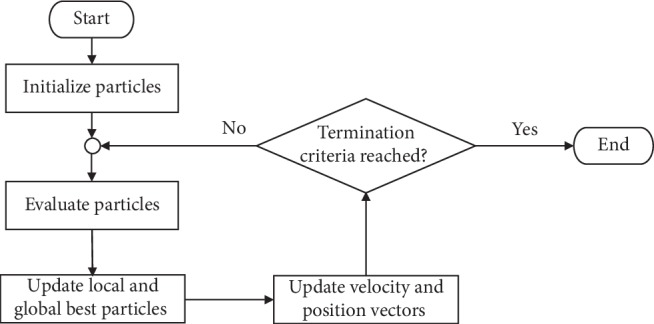
Flowchart of the PSO process.

PSO is a metaheuristic method because it makes few or no assumptions regarding the problem being optimized. A basic variant of the PSO algorithm [17, 31] works by using a population of candidate solutions (also known as particles) to explore the feasible search space. Each of these *k* particles is represented by a position vector *X*_*k*_ and a velocity vector *V*_*k*_. The movements of the particles are driven by their best-known positions Pb (local best) in addition to the entire swarm's best-known position Pg (global best) in the search space, as shown in

(2) $$\begin{array}{c} V_{k}^{d}=\omega V_{k}^{\prime d}+c_{1}r_{1}\left({\text{Pb}}_{k}^{d}-X_{k}^{\prime d}\right)+c_{2}r_{2}\left({\text{Pg}}^{d}-X_{k}^{\prime d}\right), \end{array}$$

(3) $$\begin{array}{c} X_{k}^{d}=X_{k}^{\prime d}+V_{k}^{d}, \end{array}$$

where *d* is the dimensionality of the problem, or the number of decision variables to be optimized. The PSO algorithm searches for the optimal solution in an iterative manner. In each iteration, the velocity *V* is updated by using the most recent velocity *V*′ as well as the cognitive coefficient *c*_1_ of the particle and the social coefficient *c*_2_ of the members of the swarm multiplied by random variables *r*_1_ and *r*_2_, respectively. The new position *X*_*k*_ is updated with respect to the previous position *X*_*k*_′ in accordance with the updated *V*_*k*_. A flowchart of the PSO algorithm is shown in Figure 2.

In this work, the time-varying acceleration coefficients proposed in [18] are adopted. In this version of the PSO algorithm, the cognitive coefficient *c*_1_ and the social coefficient *c*_2_ are defined to be adaptable. Beginning with a larger cognitive component and a smaller social component, the particles move around the search space instead of immediately moving toward the population's best solution. After several objective function calls, each particle has explored and collected adequate information about the search space, and the coefficients are correspondingly modified to obtain a smaller cognitive component and a larger social component to directly drive convergence to the global optimum. The modification of these two acceleration coefficients can be represented as follows:

(4) $$\begin{array}{c} c_{1}=\left(c_{1\max}-c_{1\min}\right)*\left(\frac{\text{MAXCALL}-\text{calls}}{\text{MAXCALLS}}\right)+c_{1\min},\\ c_{2}=\left(c_{2\min}-c_{2\max}\right)*\left(\frac{\text{MAXCALL}-\text{calls}}{\text{MAXCALLS}}\right)+c_{2\max}, \end{array}$$

where the maximum coefficient values *c*_1max_ and *c*_2max_ and the minimum coefficient values *c*_1min_ and *c*_2min_ are constants, calls is the most recent count of objective function calls, and MAXCALLS  is the maximum allowed number of objective function calls. Moreover, this method uses a time-varying inertial weight factor (*ω*), as shown in

(5) $$\begin{array}{c} \omega =\left(\omega_{\max}-\omega_{\min}\right)*\left(\frac{\text{MAXCALLS}-\text{calls}}{\text{MAXCALLS}}\right)+\omega_{\min}, \end{array}$$

where *ω*_max_ and *ω*_min_ are the initial and final values, respectively, of the inertial weight factors. This factor balances the local and global search capabilities during the optimization process. With a larger inertial weight factor at the beginning, the particles move more broadly and quickly around the search space. In contrast, a smaller inertial weight enables the particles to more precisely explore the search space surrounding the global optimum.

For this problem, the weights for all tools are represented in the PSO algorithm by the position vector of each particle. The New Result Vector is structured as follows:

(6) $$\begin{array}{c} \text{New Result Vector}=\left[\overset{n}{\underset{1}{\sum }}\left(w_{i},x_{i,1}\right),\overset{n}{\underset{1}{\sum }}\left(w_{i},x_{i,2}\right),\ldots ,\overset{n}{\underset{1}{\sum }}\left(w_{i},x_{i,m}\right)\right], \end{array}$$

where *w*_1_, *w*_2_,…, *w*_*n*_ are the weights for all *n* classifiers and *x*_*i*,1_, *x*_*i*,2_,…, *x*_*i*,*m*_ are the elements of the normalized result score vector corresponding to each of the *m* locations generated by each classifier used in this work.

### 2.4. Decision-Making

While the PSO algorithm is running, the New Result Vector is used to determine the protein location. Only the location with the maximum score in the matrix is determined as the final answer. Therefore, the decision rule is as follows:

(7) $$\begin{array}{c} \text{seq}⟶\text{location }k⟷\overset{n}{\underset{i}{\sum }}\left(w_{i}x_{i,k}^{\text{seq}}\right)=\underset{j}{\max}\overset{n}{\underset{i}{\sum }}\left(w_{i}x_{i,j}^{\text{seq}}\right). \end{array}$$

Later, the answer is automatically checked against the class label to evaluate the performance of the PSO weights in terms of accuracy. In our study, following the results of an empirical study by Shi and Eberhart [17], the PSO parameters were set to widely used values. *c*_1_ and *c*_2_ were set to vary over time from 2.5 to 0.5 and from 0.5 to 2.5, respectively. The inertial weight *ω* was decreased linearly from approximately 0.9 to 0.4 throughout a run. We set the number of particles to 25, and we adopted a maximum allowed number of objective functions calls of 1,000 per run as the termination criterion.

### 2.5. Software Package

The PSO-LocBact software package was developed in Python and Perl using Spyder with Python 2.7 and Perl v5.22.1. Detailed documentation is provided with the package. The program offers cross-platform compatibility. The original dataset files are included in FASTA file format. The user manual takes users through the basic usage of the software package and the settings in the configuration file for the summarization of the prediction results from other classifiers. With the guidelines provided in the user manual, users can also create and apply their own training datasets. By changing the settings in the configuration file, users can add new predictor programs and weights for their results. The PSO algorithm will consider these weights along with the probabilistic scores resulting from each predictor in the calculation of the final results. Since the software package was developed entirely in scripting languages, no additional source code is needed. Any desired modifications can be easily and freely made to the software package.

## 3. Results and Discussion

### 3.1. Predictive Performance Comparison

We assessed the performance of the 10 predictors used in this study (as summarized in Table 1).  Table 2 shows the prediction performance of each tool used in this study.  Table 2 confirms the hypothesis that some tools are better than others in predicting localization in certain compartments. Additionally, the results from each predictor are not reliable for identifying the localization of proteins in every compartment. For example, PSORTb 3.0 is the most accurate classifier, but it is not as accurate as ngLOC, CELLO, and LocTree3 in classifying cytoplasmic proteins. As another example, SLP-Local outperforms SOSUI-GramN, Gneg-mPLoc, and PSLpred for the prediction of periplasmic proteins despite its limited overall prediction competence. Similarly, despite its lack of performance in identifying cytoplasmic proteins in Gram-negative bacteria, Gpos-mPLoc (a complementary software package to Gneg-mPLoc) performs well for cytoplasmic protein samples from Gram-positive bacteria. To combine the strengths of these various predictive programs, we take advantage of PSO as a computational intelligence technique to optimize the weights associated with the different output classes for each predictive tool and combine their results to obtain a final decision. Generally, PSO has been proven to be an efficient optimization algorithm for finding an optimal solution in various fields by searching an entire multidimensional problem space. The advantages of PSO include its good robustness, simplicity, and fast convergence speed, with relatively few parameters to adjust [32–35].

As shown in Table 2, for both Gram-negative and Gram-positive bacteria, the PSO-based combination of predictors leads to a performance improvement over any single individual predictor.

### 3.2. Effect of PSO as a Combiner in PSO-LocBact

We also compared our PSO-based method with other consensus classifiers and the recently proposed single predictor called FUEL-mLoc [23]. Since no other consensus classifiers specifically designed for predicting the localization of bacterial proteins are available, various consensus classifiers using various fusion algorithms to combine a set of predictors (the set of predictors used for Gram-negative bacterial proteins consisted of CELLO, PSORTb 3.0, CELLO2GO, SOSUI-GramN, SLP-Local, ngLOC, Gneg-mPLoc, PSLpred, and LocTree3, and the set of predictors used for Gram-positive bacterial proteins consisted of CELLO, PSORTb 3.0, CELLO2GO, ngLOC, Gpos-mPLoc, and LocTree3) were implemented using the Weka machine learning workbench [36]. All consensus classifiers were trained on the training set using the 10-fold cross-validation strategy and then tested with the test sets. As shown in Table 3, our PSO-based tool shows high overall accuracy when compared with the other consensus classifiers.

Compared to the majority voting method, the PSO-based method yields increased prediction accuracies for secreted (extracellular), periplasmic, and cytoplasmic proteins in the Gram-negative bacterial protein datasets and for cell wall and extracellular proteins in the Gram-positive bacterial protein datasets. In the PSO-based method, an appropriate weight can be assigned to each class for each predictor instead of an equal weight for each predictor, which is especially important in the case of multiclass classification. Moreover, this method provides probabilistic scores indicating the confidence of the protein localization predictions. These probabilistic scores can be used to identify multiple locations of proteins. In the case of multilocation proteins, which are collocated at or move between two or more different subcellular compartments, our method is able to contribute to the simultaneous prediction of multiple subcellular locations. For individual query sequences, the predicted location with the highest score should be assigned as the most promising location of a particular protein, while the second ranking can be suggested as an alternative location for such a multilocation protein.

### 3.3. Performance of PSO-LocBact under Different Circumstances

The choice of the individual predictors considered in a consensus classifier also affects the prediction results. Since, under most circumstances, users may not know the limitations and merits of individual predictors, the aim of this section is to investigate how well PSO-LocBact performs in terms of accuracy and robustness with a limited number of predictor programs. To this end, we designed 9 experimental cases to represent various circumstances to evaluate the performance of the proposed method by removing certain programs (based on the performance results from Table 2) and then investigating the effects of this removal on the final prediction results (see Table 4). In the first experimental case, PSORTb 3.0, which achieved the highest overall accuracy, was removed from the system. With the best predictor in the list removed, the PSO classifier needs to rely on other, less efficient tools. Its overall accuracy for Gram-negative bacteria in this case is slightly decreased to 97.67% compared to the result reported for PSO-LocBact with the all-program strategy in Table 2.

As the complement to the second experimental case, the third experiment was carried out by removing all predictors with an overall accuracy higher than 90%. The predictors removed in this case for Gram-negative bacteria were PSORTb 3.0 and CELLO2GO. As shown in Table 4, our PSO-LocBact can improve the prediction performance in this case. Each predictor included in this case achieves an overall accuracy of less than 90%. By contrast, the overall prediction result of PSO-LocBact in this case is 90.69%, beyond the level attained by any of the individual predictors (CELLO, SOSUI-GramN, SLP-Local, ngLOC, Gneg-mPLoc, PSLpred, and LocTree3).

In the sixth experimental case, the predictors with overall accuracies lower than 70% were removed: CELLO, SLP-Local, Gneg-mPLoc, and PSLpred for Gram-negative bacteria and Gpos-mPLoc and LocTree3 for Gram-positive bacteria. As shown in Table 4, the results for the Gram-positive experiment in this case are even better than those of PSO-LocBact with the all-program strategy, as reported in Table 2. This finding indicates that the combination of only a few efficient tools is also adequate to produce reliable solutions.

In experimental case 9 for Gram-positive bacteria, since Gpos-mPLoc is the only classifier with an accuracy of less than 60%, we could not test our model under this condition.

Based on these 9 different experiments carried out in this study to determine the effectiveness of the PSO-LocBact method under various circumstances, we conclude that the proposed method can provide users with more confidence in the obtained predictions. These results also confirm that PSO-LocBact can increase performance and/or provide more reliable prediction results in all experimental cases. Moreover, new prediction programs can be easily added to our method; thus, any novel predictors that may be developed in the future can be easily included to further improve the prediction accuracy.

### 3.4. Comparison with State-of-the-Art Predictors and the Performance of PSO-LocBact on the Benchmark Dataset S

Note that, in our training and test datasets, we used a threshold of 90% instead of 25% sequence identity because we needed to increase the number of proteins for some classes for which only a limited number of proteins with reviewed localization statuses were available in the database in order to be able to build a balanced training dataset, which is important for building a consensus predictor. Individual homolog features are not needed to train such a model for consensus prediction, unlike most individual predictor methods, which depend on homolog features for model training and thus need to consider the homology bias of the features. In addition, we included the well-known fair benchmark dataset S, which comprises proteins that share less than 25% identity, as our validation dataset to enable performance comparisons with various state-of-the-art methods.

Table 5 shows the performance of PSO-LocBact and various state-of-the-art predictors on dataset S, which is a widely used benchmark dataset. This dataset was constructed by the authors of [7, 9] and has been used to test various predictors, including iLoc-Gneg [24], Gram-LocEN [25], Gneg-PLoc [26], Gneg-mPLoc [7], and iLoc-Gpos [27]. The overall accuracy of PSO-LocBact is 96.15% for Gram-negative bacterial proteins and 99.42% for Gram-positive bacterial proteins, higher than the values for the other state-of-the-art methods. In contrast to the dataset considered in the previous section, which is a balanced dataset, this benchmark consists of imbalanced data. Therefore, PSO-LocBact shows high performance on both balanced and imbalanced datasets.

## 4. Conclusions

With the growing number of research efforts employing various machine learning approaches to predict the subcellular localization of proteins, these tools can yield incongruent prediction results in some circumstances. In this paper, PSO-LocBact, a method of bacterial protein subcellular localization prediction based on the simple particle swarm optimization (PSO) technique, has been proposed to integrate the prediction results from preexisting predictors to provide more reliable predictions and increased accuracy under most circumstances. During testing, our proposed method achieved an overall prediction accuracy of over 98%. Hence, this method can provide researchers in the field with more reliable answers for protein localization together with probabilistic scores indicating the confidence of the results.

### 4.1. Software Package Applications

The PSO-LocBact method is a PSO method for combining the results of multiple classifiers for the prediction of protein subcellular localization in both Gram-negative and Gram-positive bacteria. This method is capable of generating final localization predictions based on protein sequence data. In particular, this method has been developed to address the inconsistency problems encountered in this task. Our recent work has focused on introducing a simple PSO method of optimizing the prediction results obtained from other applications. The software package is designed to be easy to understand and develop. In addition, users are able to use new datasets for training and testing, thus updating this software's capabilities. By modifying the configuration file, users can reconfigure the software, optimize the weights for each predictor, add more result files to aid in prediction, and even set the basic PSO parameters. These configuration variables are shown in Table 6.

## Figures and Tables

**Table 1:** Summary of predictors used in this work.

Predictor	Organism categories	Subcellular compartments predicted	URL	References
SOSUI-GramN	Gram-negative bacteria	Extracellular region, outer membrane, periplasm, inner membrane, and cytoplasm	http://harrier.nagahama-i-bio.ac.jp/sosui/sosuigramn/sosuigramn_submit.html	[1]

CELLO	Bacteria, eukaryotes	Extracellular region, outer membrane, inner membrane, periplasm, and cytoplasm	http://cello.life.nctu.edu.tw/	[2, 3]

CELLO2GO	Archaea, bacteria, eukaryotes, viruses	Extracellular region, outer membrane, inner membrane, periplasm, and cytoplasm	http://cello.life.nctu.edu.tw/cello2go/	[4]

PSLpred	Gram-negative bacteria	Extracellular region, outer membrane, inner membrane, periplasm, and cytoplasm	http://crdd.osdd.net/raghava/pslpred/	[5]

SLP-local	Prokaryotes, eukaryotes	3 locations for prokaryotes (cytoplasm, extracellular region, and periplasm)	http://sunflower.kuicr.kyoto-u.ac.jp/∼smatsuda/slplocal.html	[6]

Gneg-mPLoc	Gram-negative bacteria	Cytoplasm, extracellular region, fimbria, flagella, inner membrane, nucleoid, outer membrane, and periplasm	http://www.csbio.sjtu.edu.cn/bioinf/Gneg-multi/	[7, 8]

Gpos-mPLoc	Gram-positive bacteria	Cytoplasm, cell wall, plasma membrane, and extracellular region	http://www.csbio.sjtu.edu.cn/bioinf/Gpos-multi/	[8, 9]

PSORTb 3.0	Archaea, bacteria	4 locations for Gram-positive bacteria and archaea (cytoplasm, cytoplasmic membrane, cell wall, and extracellular region)	http://www.psort.org/psortb	[10]
		5 locations for Gram-negative bacteria (cytoplasm, inner membrane, periplasm, outer membrane, and extracellular region)		

ngLOC	Prokaryotes, eukaryotes	4 locations for Gram-positive bacteria (cytoplasm, inner membrane, cell wall, and extracellular region)	http://genome.unmc.edu/ngLOC/index.html	[11]
		5 locations for Gram-negative bacteria (cytoplasm, inner membrane, periplasm, outer membrane, and extracellular region)		

LocTree3	Archaea, bacteria, eukaryotes	3 locations for archaea (cytoplasm, extracellular region, and plasma membrane)	https://rostlab.org/services/loctree3/	[12]
		6 locations for bacteria (cytoplasm, extracellular region, fimbria, outer membrane, periplasm, and plasma membrane)		

**Table 2:** Accuracy of each individual classifier and PSO-LocBact.

Gram-negative bacterial proteins						
Location: predictor	Extracellular region	Outer membrane	Periplasm	Inner membrane	Cytoplasm	Overall

CELLO	40.69%	48.84%	76.74%	87.21%	89.53%	**68.60%**
PSORTb 3.0	100%	100%	88.37%	100%	98.84%	**97.44%**
CELLO2GO	100%	100%	87.21%	100%	100%	**97.44%**
SOSUI-GramN	66.28%	56.98%	67.44%	90.70%	87.21%	**73.72%**
SLP-local	36.05%	0	75.58%	0	65.12%	**35.35%**
ngLOC	77.91%	96.51%	86.05%	93.02%	94.19%	**89.53%**
Gneg-mPLoc	82.56%	89.53%	1.16%	100%	0	**54.65%**
PSLpred	0	100%	1.16%	0	0	**20.23%**
LocTree3	84.88%	46.51%	80.23%	93.02%	93.02%	**79.53%**
PSO-LocBact	100%	100%	94.19%	100%	100%	**98.84%**

Gram-positive bacterial proteins						
Location: predictor	Extracellular region	Cell wall	Inner membrane	Cytoplasm	Overall	

CELLO	86.84%	29.87%	100%	100%	**79.42%**	
PSORTb 3.0	93.42%	93.50%	100%	100%	**96.78%**	
CELLO2GO	97.39%	90.90%	100%	100%	**97.10%**	
ngLOC	86.84%	42.85%	93.67%	100%	**81.03%**	
Gpos-mPLoc	34.21%	24.68%	77.21%	100%	**59.81%**	
LocTree3	85.53%	0	91.14%	96.20%	**68.49%**	
PSO-LocBact	97.39%	94.80%	100%	100%	**98.07%**	

**Table 3:** Accuracy of various consensus methods on the test sets.

Gram-negative bacterial proteins						
Location:	Extracellular region (%)	Outer membrane (%)	Periplasm (%)	Inner membrane (%)	Cytoplasm (%)	Overall (%)

Single predictors (as shown in Table 2)	0–100	0–100	1.16–88.37	0–100	0–100	**20.23–97.44**
Consensus classifier: PSO-LocBact	100	100	94.19	100	100	**98.84**
Consensus classifier: majority voting	97.67	100	95.35	100	98.84	**98.37**
Consensus classifier: Naïve Bayes	100	98.84	94.18	100	98.84	**98.37**
Consensus classifier: logistic regression	98.84	100	97.67	95.35	98.84	**98.14**
Consensus classifier: average probability voting	98.84	100	90.69	98.84	98.84	**97.44**
Single predictor: FUEL-mLoc (2017)	79.07	97.67	96.51	93.02	82.56	**89.76**

Gram-positive bacterial proteins						
Location:	Extracellular region (%)	Cell wall (%)	Inner membrane (%)	Cytoplasm (%)	Overall (%)	

Single predictors (as shown in Table 2)	34.21–97.39	0–93.50	77.21–100	97.50–100	**59.81–97.10**	
Consensus classifier: PSO-LocBact	97.39	94.80	100	100	**98.07**	
Consensus classifier: majority voting	93.42	93.50	100	100	**96.78**	
Consensus classifier: Naïve Bayes	69.73	92.20	100	100	**90.67**	
Consensus classifier: logistic regression	89.47	100	100	100	**97.43**	
Consensus classifier: average probability voting	96.05	87.01	100	98.73	**95.49**	
Single predictor: FUEL-mLoc (2017)	86.84	81.82	100	100	**92.28**	

**Table 4:** Accuracy of PSO-LocBact in different experimental cases.

Gram-negative bacterial proteins						
Location:	Extracellular region (%)	Outer membrane (%)	Periplasm (%)	Inner membrane (%)	Cytoplasm (%)	Overall (%)

Experimental case 1	98.84	100	93.02	97.67	100	**97.67**
Experimental case 2 (>90)	100	100	87.21	100	100	**97.44**
Experimental case 3 (<90)	80.23	91.86	90.69	94.19	96.51	**90.69**
Experimental case 4 (>80)	100	100	89.53	100	98.84	**97.67**
Experimental case 5 (<80)	84.88	54.65	84.88	95.35	94.19	**82.79**
Experimental case 6 (>70)	100	100	94.19	100	100	**98.84**
Experimental case 7 (<70)	44.19	66.28	81.4	88.37	95.35	**75.12**
Experimental case 8 (>60)	100	100	94.19	100	98.83	**98.60**
Experimental case 9 (<60)	76.74	96.51	86.05	81.39	93.02	**86.74**

Gram-positive bacterial proteins						
Location:	Extracellular region (%)	Cell wall (%)	Inner membrane (%)	Cytoplasm (%)	Overall (%)	

Experimental case 1	96.05	100	100	98.73	**98.71**	
Experimental case 2 (>90)	96.34	96.39	100	100	**98.20**	
Experimental case 3 (<90)	89.02	54.22	98.82	100	**85.63**	
Experimental case 4 (>80)	97.56	96.39	100	100	**98.50**	
Experimental case 5 (<80)	78.05	43.37	91.76	100	**78.44**	
Experimental case 6 (>70)	94.73	100	100	100	**98.71**	
Experimental case 7 (<70)	68.29	12.05	100	100	**70.66**	
Experimental case 8 (>60)	97.56	96.39	100	100	**98.50**	
Experimental case 9 (<60)	NA	NA	NA	NA	**NA**	

Experimental case 1: performance of PSO-LocBact without PSORTb 3.0. Experimental case 2: performance of PSO-LocBact considering only classifiers with accuracy ≥90%. Experimental case 3: performance of PSO-LocBact considering only classifiers with accuracy <90%. Experimental case 4: performance of PSO-LocBact considering only classifiers with accuracy ≥80%. Experimental case 5: performance of PSO-LocBact considering only classifiers with accuracy <80%. Experimental case 6: performance of PSO-LocBact considering only classifiers with accuracy ≥70%. Experimental case 7: performance of PSO-LocBact considering only classifiers with accuracy <70%. Experimental case 8: performance of PSO-LocBact considering only classifiers with accuracy ≥60%. Experimental case 9: performance of PSO-LocBact considering only classifiers with accuracy <60%.

**Table 5:** Accuracy of PSO-LocBact compared to other state-of-the-art methods on the well-known benchmark dataset S taken from [7, 9, 30].

Gram-negative bacterial proteins						
Benchmark dataset S: predictor	Inner membrane (557 proteins)	Outer membrane (124 proteins)	Cytoplasm (410 proteins)	Extracellular region (133 proteins)	Periplasm (180 proteins)	Overall (1,404 proteins)

PSO-LocBact	547	116	387	129	171	**1,350 (96.15%)**
Gram-LocEN [25]	551	116	374	130	169	**1,340 (95.44%)**
PSORTb 3.0 [10]	529	114	380	117	168	**1,308 (93.16%)**
CELLO2GO [4]	519	107	383	128	170	**1,307 (93.09%)**
Gneg-PLoc [26]	454	68	362	59	87	**1,030 (73.36%)**
Gneg-mPLoc [7]	525	105	357	79	154	**1,220 (86.89%)**
iLoc-Gneg [24]	539	103	367	115	161	**1,285 (91.52%)**
Fuel-mLoc [23]	541	111	379	129	161	**1,321 (94.09%)**

Gram-positive bacterial proteins						
Benchmark dataset S: predictor	Cell membrane (174 proteins)	Cell wall (18 proteins)	Cytoplasm (208 proteins)	Extracellular region (123 proteins)	Overall (523 proteins)	

PSO-LocBact	174	18	206	122	**520 (99.42%)**	
Gram-LocEN [25]	173	17	203	120	**513 (98.08%)**	
PSORTb 3.0 [10]	169	14	203	112	**498 (95.22%)**	
CELLO2GO [4]	149	10	197	121	**477 (91.2%)**	
iLoc-Gpos [27]	167	12	198	110	**487 (93.12%)**	
Fuel-mLoc [23]	170	17	202	117	**506 (96.75%)**	
Gpos-PLoc [30]	—	—	—	—	**379 (72.47%)**	
Gpos-mPLoc [9]	—	—	—	—	**430 (82.22%)**	
ML-KNN [28]	—	—	—	—	**78.71%**	
wML-KNN [29]	—	—	—	—	**91.49%**	

**Table 6:** PSO-LocBact configuration variables.

Configuration variable	Value type	Default value	Description
w1	Float	0.9	Inertial weight value at the beginning of PSO
w2	Float	0.4	Inertial weight value at the end of PSO
c1i	Float	2.5	Cognitive coefficient value at the beginning of PSO
c1f	Float	0.5	Cognitive coefficient value at the end of PSO
c2i	Float	0.5	Social coefficient value at the beginning of PSO
c2f	Float	2.5	Social coefficient value at the end of PSO
Particle num	Integer	25	Number of particles generated in the swarm
MAXOBJ	Integer	1,000	Maximum number of allowable objective function calls
MAXITER	Integer	—	Maximum number of allowable iterations; if this value is set, MAXOBJ will be ignored
(Program_name)	String	(Gram-negative: CELLO, PSORTb 3.0, CELLO2GO, SOSUI-GramN, SLP-Local, ngLOC, Gneg-mPLoc, PSLpred, LocTree3; Gram-positive: CELLO, PSORTb 3.0, CELLO2GO, ngLOC, Gpos-mPLoc, LocTree3)	A list of names of the programs used to calculate the final result
(Weight)	Float		A list of weights given to represent the reliability of every program included

## Data Availability

The training and test datasets supporting the analysis in this study are from previously reported studies and datasets, which have been cited. The software is available from the corresponding author upon request. http://www.ncrna-pred.com/psolocbact.html.
